# Current Practices and Priorities of Anesthetists and Consumers for Infants Undergoing Inguinal Hernia Surgery

**DOI:** 10.1111/pan.70060

**Published:** 2025-09-30

**Authors:** Fiona J. Taverner, Bojana Stepanovic, Claire T. Roberts, Britta S. von Ungern‐Sternberg, Scott Morris

**Affiliations:** ^1^ Flinders University, College of Medicine and Public Health Adelaide South Australia Australia; ^2^ Department of Anaesthesia and Pain Management Southern Adelaide Local Health Network Adelaide South Australia Australia; ^3^ Women's and Children's Health Network Adelaide South Australia Australia; ^4^ Department of Anaesthesia and Pain Medicine Perth Children's Hospital Nedlands Western Australia Australia; ^5^ Institute for Paediatric Perioperative Excellence The University of Western Australia Perth Western Australia Australia; ^6^ Division of Emergency Medicine, Anaesthesia and Pain Medicine The University of Western Australia Perth Western Australia Australia; ^7^ Perioperative Medicine Team, Perioperative Care Program The Kids Research Institute Australia Nedlands Western Australia Australia; ^8^ Neonatal Unit, Southern Adelaide Local Health Network Adelaide South Australia Australia

**Keywords:** general anesthetics, gestational age, inguinal hernia, neonatal intensive care units, newborn infant, premature infant

## Abstract

**Introduction:**

There is a paucity of data on the chosen anesthesia management for infant inguinal hernia surgery. We aimed to characterize self‐reported anesthetic practice in Australia and New Zealand. We also aimed to identify the outcomes that matter to both anesthetists and to parents and carers.

**Methods:**

Two separate surveys were administered, one for anesthetists and the other for parents and carers of infants who had undergone an inguinal hernia operation.

**Results:**

Eighty‐nine surveys were completed by anesthetists. The most common preferred anesthetic technique was general anesthesia for both preterm 55 (61.8%) and term infants 79 (91%). Anesthetists reported that an infant's gestational age at birth and health factors influence their choice of anesthetic, as well as their personal and institutional experience. The highest priority of anesthetists was “avoiding intraoperative critical events.” Eighty‐three surveys were completed by parents or carers. 48 (57.8%) reported their infant born preterm, and 50 (62.7%) spent time in the neonatal intensive care unit. The highest ranked priority for parents was “minimising impact on baby's brain.”

**Conclusion:**

The most common anesthetic type reported by parents and anesthetists for both preterm and term infants undergoing inguinal hernia surgery in Australia and New Zealand is general anesthesia. Many anesthetists do not feel confident in performing neuraxial blocks in infants; however, regional techniques are more likely to be preferred for preterm infants. In this study, anesthetists' top‐ranked priority was avoiding intraoperative critical events, and parents and carers' top‐ranked priority was minimizing impact on their baby's brain. Differences in the priorities of anesthetists and consumers indicate the need to ensure clinicians discuss issues relevant to parents in preoperative counseling. The need to involve consumers in future research directions and study design is highlighted. Data on anesthetic outcomes following hernia surgery in an Australian and New Zealand context are needed.

## 1 Introduction

Inguinal herniotomy is one of the most common surgical procedures in infants [1]. It is of particular interest to many clinicians involved in the perioperative care of these infants due to the higher rate of prematurity and the consequent higher likelihood of comorbidities and inherent risks associated with anesthesia [1].

In Australia and New Zealand, a recent three‐centre pilot study of caudal block, high‐flow nasal oxygen insufflation, and dexmedetomidine sedation has shown the potential of an awake regional anesthetic bundle to avoid general anesthesia with an acceptable perioperative complication rate [2]. However, there is a lack of information on what the most common anesthetic techniques are for infant inguinal herniotomy in Australia or New Zealand. Recently, the NECTARINE group reported that in Europe over 85% of infants receive general anesthesia (with or without regional) and under 15% are managed with an awake regional technique [3]. Surgeons of the European Pediatric Surgeons Association reported that 54% favor an open approach under general anesthesia when operating on preterm infants [4]. The authors were unable to find this information reported for any other region.

There are also few available data regarding anesthetist and consumer (parent or carer) concerns or priorities for these infants. One survey that focused specifically on neurodevelopment showed that 49% of parents were moderately or very concerned about their infant undergoing anesthesia, and 40% were concerned about neurodevelopment [5]. The authors cannot find any other studies that focused on anesthetist or consumer priorities for infants undergoing inguinal hernia surgery.

Establishing current anesthetic approaches to herniotomy in Australia and New Zealand, as well as an understanding of anesthetist priorities, is needed to determine the drivers for current practice and the barriers to implementing promising newer techniques, such as caudal, high‐flow nasal oxygen insufflation, and dexmedetomidine sedation. Furthermore, consumer priorities need to be understood to guide quality improvement and future research directions [6, 7].

This study aimed to survey Australian and New Zealand anesthetists to determine how anesthesia is provided during infant hernia surgery. We also aimed to survey the priorities of anesthetists when delivering anesthesia during infant herniotomy and to retrospectively survey parents and carers of infants undergoing inguinal hernia surgery to determine consumer priorities as a basis for future patient and family‐centred research into novel anesthetic approaches to infant hernia surgery.

## 2 Methods

Ethics approval was granted by the Women's and Children's Hospital Human Research Ethics Committee (approval number 2022/HRE00141). Two separate surveys were administered, one for anesthetists and the other for parents and carers. The first page of the electronic survey contained participant information. Voluntary completion of the survey following this information implied consent to participate. Development and dissemination of these surveys are outlined below.

### 2.1 Anesthetist Survey

The target population for the anesthetist survey was anesthetists who have looked after infants undergoing inguinal hernia surgery. In Australia and New Zealand, these anesthetists would often have undergone additional training in neonatal and pediatric anesthesia and would regularly be involved in the care of pediatric patients.

The anesthetist survey (detailed in Appendix S1) was sent to the Society of Pediatric Anesthesia in New Zealand and Australia (SPANZA) members via a link in the members' newsletter, which is distributed by email to all current members (406 members in 2022), as well as the display of a QR code at the SPANZA Annual Scientific Meeting in October 2022. Responses were anonymous. The survey included questions about anesthetic preferences for infants undergoing herniotomy, asked respondents to rank priorities for anesthesia outcomes, and what reduction in postoperative intubation rate with a new anesthetic technique would change their practice.

### 2.2 Consumer Survey

The target population for this retrospective survey (detailed in Appendix S2) was the parents and carers of infants who had undergone an inguinal hernia operation. Responses were anonymous. A consumer advisor assessed the survey to ensure that the language used was appropriate for the target population and assisted in matching the parent priorities list to the priorities listed in the anesthetist survey to allow comparison of the responses between the anesthetists and consumers.

The survey was emailed to parents and carers of infants who had an inguinal hernia operation at the Women's and Children's Hospital, Adelaide, Australia after they had been discharged from hospital, from January 2020 to October 2023. The survey was also disseminated via a direct link to the survey on the Miracle Babies Foundation social media site and the “Parents Who Have Been There” Facebook group; these two parent‐founded charitable organizations are Australia‐wide and state‐wide in South Australia respectively and widely used to engage consumers in research design and implementation. Using social media advertising to promote the survey via these groups enabled an Australia‐wide consumer survey sample to match the Australia‐wide SPANZA survey sample. New Zealand consumers were not, however, included as Miracle Babies is solely an Australian‐based foundation.

### 2.3 Data Analysis

Surveys were created and results collated in a REDCap database. Descriptive statistics were performed for quantitative data. The paired prioritization was analyzed by weighting each ranked answer, where rank 1 was assigned a score of 3, rank 2 assigned a score of 2, rank 3 assigned a score of 1, and no ranking scored 0. The averages of these scores were then analyzed using the Mann Whitney U test, and the Glass rank biserial correlation co‐efficient [8] was used to calculate the standardized effect size using IBM SPSS statistics software, version 30.0.0.0 (172). Qualitative data (free text survey responses) were analyzed by grouping the comments into themes by two independent team members (FT and BS).

## 3 Results

### 3.1 Anesthetist Survey

Eighty‐nine surveys were completed. Respondents reported a principal workplace location in every state and territory in Australia and New Zealand, apart from the Northern Territory, as shown in Table 1.

**TABLE 1:** SPANZA anesthetist workplace location.

SPANZA anesthetist workplace location	*N* (%)
New South Wales and Australian Capital Territory	9 (10.1%)
Queensland	16 (18%)
South Australia	7 (7.9%)
Tasmania	2 (2.2%)
Victoria	10 (11.2%)
Western Australia	26 (29.2%)
New Zealand	17 (19.1%)
Not answered	2 (2.2%)

Full details of anesthetists' reported preferred anesthetic technique are listed in Table 2.

**TABLE 2:** Anesthetists preferred anesthetic technique for preterm and term infants.

Preferred anesthetic technique	Preterm	Term
General anesthetic with caudal anesthetic	45 (50.6%)	63 (70.8%)
General anesthetic without caudal anesthetic	7 (7.9%)	16 (18%)
Spinal anesthetic	28 (31.5%)	4 (4.5%)
Other	9^a^ (10.1%)	6^b^ (6.7%)

^a^ Other anesthetic techniques listed by respondents for preterm infants are as follows: spinal anesthetic and caudal anesthetic (1); caudal anesthetic with sedation (2); caudal anesthetic only (2); rarely do preterm infants (1); general anesthetic and ilioinguinal block by surgeon (1); general anesthetic with local anesthetic to wound by surgeon (2).

^b^ Other anesthetic techniques listed by respondents for term infants are as follows: general anesthetic with ilioinguinal block for unilateral hernias, general anesthetic and caudal for bilateral hernias (1); general anesthetic and ilioinguinal block by surgeon (1); caudal anesthetic with sedation (1); caudal anesthetic only under 44 weeks postmenstrual age, otherwise general anesthetic with caudal anesthetic (1); general anesthetic with local anesthetic to wound by surgeon (2).

The percentage change in postoperative intubation rate for a new anesthetic technique which would convince anesthetists to change their practice is reported in Table 3.

**TABLE 3:** The percentage change in post operative intubation rate for a new anesthetic technique which would convince anesthetists to change their practice.

Percentage change required to change practice	Anesthetist response *n* (%)
2%	14 (15.7%)
4%	15 (16.9%)
6%	31 (34.8%)
9%	15 (16.9%)
Current postoperative intubation rate is acceptable, would not change practice	12 (13.5%)

**FIGURE 1:**
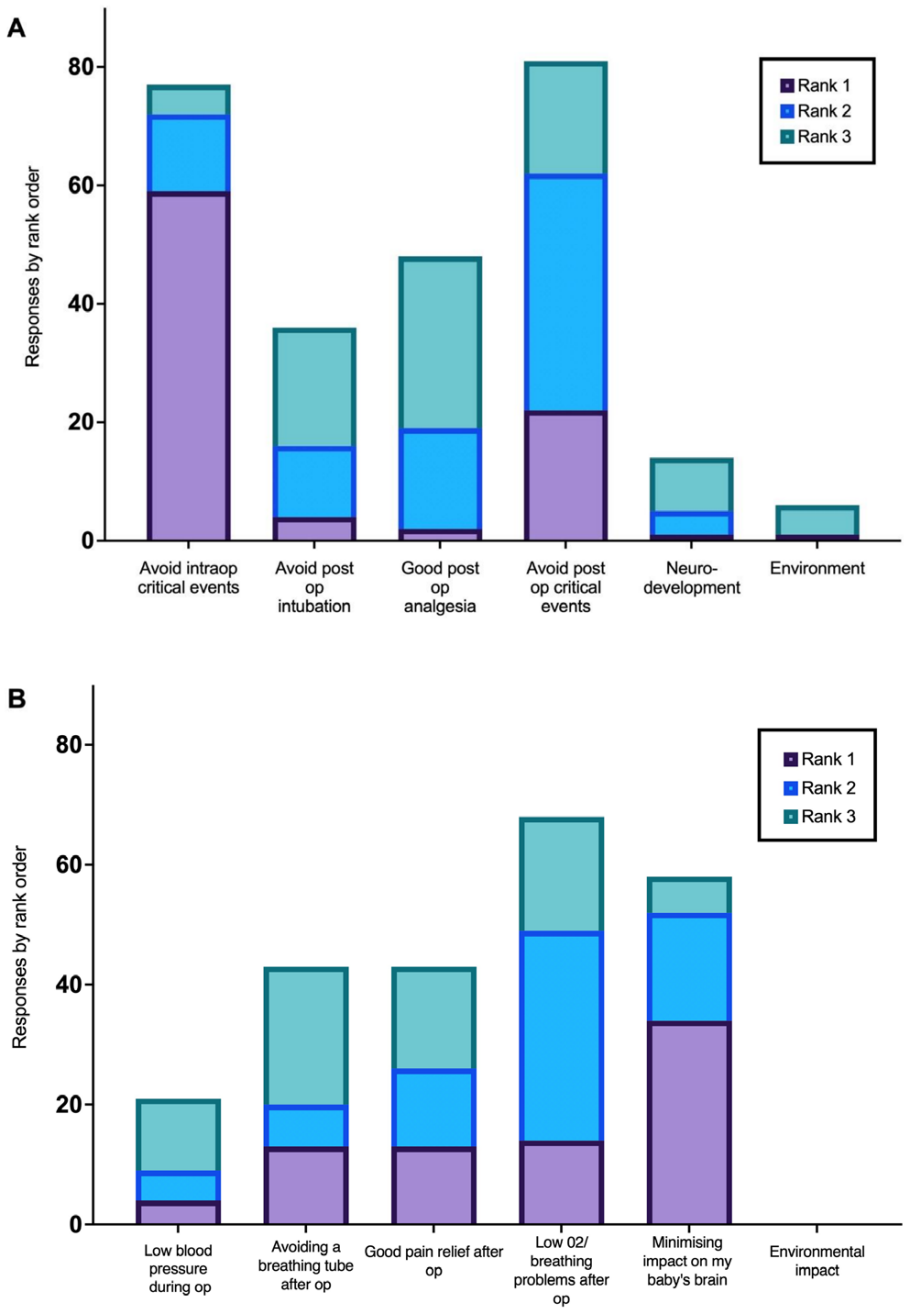
(A) Anesthetist responses to priorities by ranking. (B) Parent and carer responses to priorities by ranking. Rank 1 is the highest ranked priority for each respondent, rank 2 is the second ranked, rank 3 is the third rank. No responses were recorded for lower ranked priorities.

The anesthetist's responses to ranking their priorities for an infant are displayed in Figure 1 and the average scores of their responses are shown in Table 4.

**TABLE 4:** Average scores of anesthetists compared to parents and carers for their ranked priority responses.

	Anesthetist average score	Parents and carer average score	*p value*	Standardized effect size, correlation co‐efficient
Avoiding low blood pressure during the operation	2.34	0.41	< 0.001	0.75
Avoiding a breathing tube after surgery	0.63	0.91	0.92	−0.13
Good pain relief after surgery	0.78	0.99	0.441	0.06
Avoiding low O_2_ levels or breathing problems after surgery	1.85	1.58	0.68	0.15
Minimizing impact on my baby's brain	0.44	1.73	< 0.001	−0.62
Environmental impact of anesthetic technique	0.09	0	0.016	0.07

*Note:* A score of 3 corresponds with the highest priority, and a score of 0 corresponds with the lowest priority.

Anesthetists were given the opportunity for free text comments at the end of the survey. These comments were grouped into three major themes: factors affecting their selection of anesthetic technique, attitudes toward neuraxial techniques, and perception of the reintubation rate post general anesthesia being lower than quoted in the survey.

#### 3.1.1 Theme 1: Factors Affecting Anesthetists Selection of Anesthetic Technique

Some anesthetists commented that they, or the entire group of specialists in the department, had de‐skilled in spinal techniques due to low volume of practice, but that nevertheless they felt general anesthesia techniques were managed safely with low re‐intubation rates. Anesthetists quoted a wide range of patient, anesthetic, surgical, and logistical factors that impacted their choice of spinal versus general anesthesia technique. Patient factors included weight, degree of prematurity, respiratory disease, other comorbidities, and antenatal course. Anesthesia factors included personal preference or comfort in performing a technique, training, recency of practice, and volume of practice in spinal techniques, the ability to use alternative modalities safely such as general anesthesia and caudal or general anesthesia and inguinal nerve block, and opioid‐free anesthesia. Surgical factors included hernia size, unilateral versus bilateral surgery, and surgical preference. Pertinent quote: “I don't do ex‐prems in my hospital, but if I did, the rate of post‐op reintubation is not the only consideration that would guide my practice. Personal volume of practice, surgical duration and institutional comfort with spinals would be far more important considerations for me.”

#### 3.1.2 Theme 2: Attitudes Toward Neuraxial Techniques

Some anesthetists strongly favored a spinal technique for neonatal hernia repair. Those who are proficient, experienced, and have a regular practice of spinal techniques feel very comfortable performing them. However, some anesthetists felt less skilled in performing spinal techniques due to limited practice individually and within their institutions and felt significant barriers to developing skills in neonatal spinal anesthesia. Some anesthetists opted for spinal anesthesia as a first choice for higher‐risk neonates with a history of prematurity, a complicated preoperative course, and a history of respiratory illness including apneas or requirement for respiratory support.

#### 3.1.3 Theme 3: Perception of the Reintubation Rate Quoted in the Survey

Many anesthetists surveyed felt that the quoted 9%–16% postoperative intubation rate for neonates undergoing hernia repair under general anesthesia was much higher than at their institution or in their clinical experience. They questioned where this data arose, the population studied, and whether the 9%–16% figure included late re‐intubation in the neonatal intensive care unit rather than immediately postoperatively. Pertinent quote: “I think our current postop intubation rate for hernia repairs is lower than the suggested low number in the choices, so ‘the current GA rate is acceptable’ part seems true but the ‘9%–15%’ seems untrue in our practice.”

### 3.2 Parent and Carer Survey

Eighty‐three surveys were completed. Parent and carer reported gestational age at birth of their infant, anesthetic type, and their satisfaction with their child's anesthetic are reported in Table 5.

**TABLE 5:** Parent and carer reported anesthetic technique, history of neonatal intensive care unit (NICU) admission, and satisfaction.

	*N* (%)
Gestational age at birth	
< 27 weeks	18 (22%)
27–32 weeks	15 (18%)
32–36 weeks	15 (18%)
≥ 37 weeks	35 (42%)
History of NICU admission	52 (62.7%)
Anesthetic type as recalled by parents and carers	
General anesthetic	55 (66.2%)
Spinal anesthetic	8 (9.6%)
Baby CHiX^a^	2 (2.4%)
Not sure	18 (21.7%)
Parental reported satisfaction with anesthetic	
Very satisfied	45 (54.2%)
Satisfied	26 (31.3%)
Neutral	9 (10.8%)
Dissatisfied	2 (2.4%)
Very dissatisfied	1 (1.2%)

^a^ Baby CHiX technique = caudal, high‐flow nasal oxygen and dexmedetomidine sedation.

The parents' responses to ranking their priorities for their child's anesthesia are displayed in Figure 1B and the average scores of their responses are shown in Table 4.

When parents and carers were asked about the satisfaction of their infant's anesthetic, they were grouped into the following themes: the parents' experience of communication by anesthesia/healthcare staff, parental worries or fears regarding anesthesia, and their positive and negative experiences of the process.

#### 3.2.1 Theme 1: Communication by Anesthesia/Health Care Staff

Many parent and carer comments stated that the communication by the anesthetist and surgical team was clear and effective and highly satisfactory. The communication was described as “well explained” and “compassionate and empathetic.”

#### 3.2.2 Theme 2 Parental Worries Regarding Anesthesia

Parental concerns regarding the anesthetic were sub‐grouped into three areas:

- Concern around pain and distress from procedures, needles, the IV cannula, being held down and distress from being held by strangers.
- Concern about not being able to come off a ventilator post general anesthesia.
- Concern about long‐term consequences of anesthesia drugs, concern about adverse effects for the child later in life.

Some parents and carers commented that they were happy with their child's quick recovery, good pain control, and stability post anesthesia/surgery. Others described negative experiences associated with their child's anesthesia/surgery related to unexpected complications including excessive post‐operative sedation, unexpected ICU transfer, and nasal trauma from nasal prong oxygen.

Parents and carers were asked to comment on any questions they wished they had asked prior to their child's anesthetic. These answers varied greatly. On one end of the spectrum, many felt they were thoroughly informed, had in‐depth explanations by their anesthetists, and had no further questions. Some were very comfortable in deferring decisions to the anesthetist as the “Anesthetist knows best.” Other parents and carers felt that the risks of anesthesia were not discussed or not discussed in enough detail. A small proportion of parents wanted to know more about the risks of severe adverse reactions and death post anesthesia and wanted to know the detailed incidence of risks. One response was, “What is the chance my baby is going to have a cardiac arrest and can't be revived despite the best effort and modern medical care, like percentage out of 100%, there are always a few percent of bad statistics that doctors of course don't want to mention for some reason.” Some also wanted to know about the risks of long‐term effects of general anesthesia on their child.

## 4 Discussion

In this study we describe current self‐reported anesthesia practice for inguinal hernia surgery in Australia and New Zealand, and anesthetist and parent priorities for anesthesia in caring for these infants. The most common technique preferred by anesthetists was general anesthesia (with or without caudal) for both preterm 55 (61.8%) and term infants 79 (91%). Anesthetist and consumer priorities differ greatly, with anesthetists' top priority avoiding intraoperative critical events, and parents and carers' top priority being neurodevelopment.

General anesthesia is currently more common than spinal anesthesia or other regional‐based techniques in Australia and New Zealand based on the survey results, and this is consistent with European data [3]. Nevertheless, most anesthetists did report a preference for incorporating some regional anesthesia, either as a spinal or caudal adjunct to general anesthesia. For preterm infants, the lower reported preference by anesthetists for general anesthesia, and consequently increased preference for a regional anesthesia technique, suggests that gestational age at birth (and perhaps by proxy perioperative risk) significantly influences the selection of anesthetic technique. Anesthetists commented that many factors should be considered when selecting the anesthetic type for an infant, which are not solely dependent on the infant, but also on anesthetic experience and surgical factors. Respondents who selected “other” for their preferred anesthetic technique in Table 2 quoted either gestational age at birth or surgical factors (unilateral or bilateral) as a reason for their choice. These additional elements, such as anesthetic experience, surgical, and institutional factors, may contribute to the prevalence of general anesthesia use despite the higher recognized complication rates [9, 10] and ongoing concerns regarding its impact on neurodevelopment [11] compared to spinal or other regional techniques.

The majority of anesthetists responded that a 6% or less threshold for postoperative intubation for a new anesthetic technique would convince anesthetists to change their practice. However, many anesthetists commented that they disagreed with the quoted figure of 9%–16%, which was at least not accurate in their hands or at their institution. These figures were taken from a 2018 retrospective cohort study of 263 ex‐premature neonates in the United States undergoing inguinal hernia surgery showing an overall postoperative ventilator dependence of 8.3%, and 14.7% in babies whose operation occurred prior to discharge [12], and a 2019 metanalysis of 4 randomized controlled trials and 2 case–control studies from 2000 to 2018 which compared spinal anesthesia to general anesthesia in regard to intraoperative and postoperative outcomes, reporting that 13% of infants required postoperative mechanical ventilation with general anesthesia [10]. The anesthetist's perception highlights the potential disparity, or lack of translatability, between clinical practice and the current reported research literature. These findings also highlight the need for more comprehensive multi‐institutional outcome data in an Australian and New Zealand context, which at present is lacking.

The priorities of anesthetists and parents vary greatly. 58 (69.9%) parents rated neurodevelopment concerns in their top 3 priorities for their infants, consistent with a previous study that reported 40% of parents were concerned about neurodevelopment following anesthesia [5]. In contrast, anesthetists infrequently ranked neurodevelopment in their top 3. Anesthetists ranked “avoid intraoperative critical events” highest, yet few parents prioritized “avoiding low blood pressure during the operation.” These differences may reflect parents' focus on outcomes that they can directly experience, such as postoperative pain, the need for postoperative intubation, and long‐term issues of neurodevelopment. In contrast, anesthetists may prioritize intraoperative critical events because they are responsible for acute care during the procedure. The large disparity may also be due to the findings of the GAS study and other recent studies largely reassuring anesthetists that general anesthesia has little effect on neurodevelopment and allay concerns of anesthetists [11, 13]. The difference in priorities reflects a gap in understanding risks and concerns and highlights a need for more comprehensive education and two‐way communication.

There was, however, agreement between parents and anesthetists for some priorities, avoiding postoperative critical events scored highly for both groups, and similarly, the environmental impact of the anesthetic technique scored poorly for both groups. This highlights that some priorities of care for these infants are aligned. There has been significant interest in recent years by both the community and anesthetists specifically in improving environmental sustainability and practice, but it is important to note that this priority does not rank highly when considering a high‐risk population [9].

There are several strengths to this study. The anesthetist survey sampled all states and territories in Australia except for the Northern Territory and included the whole of New Zealand. The key anesthetist and consumer questions relating to priorities for infant care during anesthesia were harmonized to allow comparison. The consumer survey was also Australia‐wide, and both surveys were contemporaneous. The data are novel in addressing a knowledge gap in the literature regarding consumer concerns for their infants undergoing inguinal hernia surgery. The infant demographics reported by parents and carers, such as gestational age at birth and requirement for a neonatal intensive care unit, are similar to other studies of infants undergoing inguinal hernia surgery, suggesting the parental responses have external validity [2, 13].

The limitations of this study design are that the parents and carers survey was only offered in English in an electronic format; therefore, some groups such as those with English as a Second Language and those with social disadvantage or limited access to electronic devices are less likely to have responded to this survey. The survey was predominantly distributed to parents and carers likely to reside in South Australia and the Northern Territory (as the catchment area of the WCH); therefore, the concerns raised by these parents may not be representative of other regions. The dissemination of the consumer survey through the Australia‐wide Miracle Babies group may minimize this bias. The anesthetists surveyed were from a larger geographic spread around Australia and New Zealand, which is a strength overall, but differs from the consumer population. The anesthetist survey response rate was 22% of all SPANZA members, which may limit generalizability; however, not all SPANZA members would be involved in the care of infants undergoing hernia surgery. The simple nature of the survey (aiming to increase the response rate) means that some anesthetists commented that not enough detail was given in the questions to be able to determine their preferred anesthetic type. The paired anesthetists and consumer ranking using an assigned score has an inherent assumption that there is linear prioritization of rankings, which may not be the case.

Future studies should explore the reasons for the discrepancy in concerns by clinicians and parents as a basis for improving educational resources. This study highlights the need for future research directions to involve consumers, to ensure that outcomes being measured are patient and parent‐centered and are as meaningful for them as they are for clinicians. Future studies could also explore anesthetists' personal and institutional barriers to performing spinal or other regional anesthesia techniques and how these barriers can be overcome.

## 5 Conclusion

The most common anesthetic type reported by parents and anesthetists for both preterm and term infants undergoing inguinal hernia surgery in Australia and New Zealand is general anesthesia. Many anesthetists do not feel confident in performing neuraxial blocks in infants; however, regional techniques are more likely to be preferred for preterm infants. Anesthetists report that an infant's gestational age at birth and health factors influence their choice of anesthetic, as well as their personal and institutional experience. The priorities of anesthetists and parents and carers vary greatly, with anesthetists' top‐ranked priority of avoiding intraoperative critical events and parents' and carers' top‐ranked priority focused on protecting their baby's brain. This difference indicates the need to ensure clinicians discuss issues relevant to consumers in preoperative counseling. The need to involve consumers in future research directions and study design is also highlighted. Data on anesthetic outcomes following hernia surgery in an Australian and New Zealand context are needed.

## Supporting information


**Appendix S1:** Anesthetist Survey Questions

**Appendix S2:** Parent and Carer Survey Questions

## Data Availability

The data that support the findings of this study are available on request from the corresponding author. The data are not publicly available due to privacy or ethical restrictions.
