# Supplementary material for: Current Practices and Priorities of Anesthetists and Consumers for Infants Undergoing Inguinal Hernia Surgery
Source: Paediatr Anaesth. 2025 Sep 30;35(12):1046–52. doi: 10.1111/pan.70060 (PMC12603882; doi:10.1111/pan.70060)
Supplement: Supplementary file 1 — Appendix S1: Anesthetist Survey Questions [file PAN-35-1046-s001.docx]

**Appendix 1:** Anesthetist survey questions

1. What is your principal workplace location? 0

South Australia

New South Wales/ ACT

Victoria

Northern Territory

Tasmania

Western Australia

New Zealand

Queensland

Question Title

* 2. Do you anesthetise neonates or infants for hernia surgery? 0

Yes

No

Question Title

* 3. Have you done a spinal in the last 12 months for neonate/infant

inguinal hernia surgery? 0

Yes

No

* 4. Have you done a caudal in the last 12 months for neonate/infant

inguinal hernia surgery? 0

Yes

No

* 5. What is your preferred anesthetic technique for exprems

undergoing hernia surgery? 0

Spinal

GA + caudal

GA without caudal

Other (please specify)

* 6. What is your preferred technique for term infants undergoing

hernia surgery? 0

Spinal

GA + caudal

GA

Other (please specify)

* 7. What are your top 3 goals when anesthetising a neonate/infant for

inguinal hernia surgery? 0

Avoiding intraoperative critical events (hypoxaemia, bradycardia,

hypotension, apnoea)

Avoiding postoperative intubation

Good postoperative analgesia

Avoiding postoperative critical events (hypoxaemia, bradycardia,

hypotension, apnoea)

Minimising impact on neurodevelopment

Environmental impact of the anesthetic technique

* 8. When doing a caudal in infants/ neonates how often do you use

ultrasound? 0

Routinely

Occasionally

Never

I don't do caudals

* 9. The literature reports a 9 - 15% post operative intubation rate

for ex prem neonates undergoing general anesthesia for inguinal

hernia surgery.

If a new technique had a lower post operative intubation rate, what

rate would be needed to consider changing your practice? 0

2% or less

4% or less

6% or less

9% or less

The current GA rate is acceptable, I wouldn't change my practice

Comments (optional)
