# Supplementary material for: Current Practices and Priorities of Anesthetists and Consumers for Infants Undergoing Inguinal Hernia Surgery
Source: Paediatr Anaesth. 2025 Sep 30;35(12):1046–52. doi: 10.1111/pan.70060 (PMC12603882; doi:10.1111/pan.70060)
Supplement: Supplementary file 2 — Appendix S2: Parent and Carer Survey Questions [file PAN-35-1046-s002.docx]

**Appendix 2:** Parent and carer survey questions

* 1. Did your baby have an operation for an inguinal hernia when they were under 1

year old? 0

Yes

No

* 2. How many weeks into their pregnancy was your baby when they were born? 0

less than 27 weeks

27 - 32 weeks

32 - 36 weeks

more than 37 weeks

* 3. Did your baby ever spend time in a neonatal intensive care unit or special care

unit? 0

Yes

No

Not sure

* 4. What kind of anesthetic did your baby have? 0

Spinal

Baby CHiX

General anesthetic

I don't know

* 5. How satisfied were you with your baby's anesthetic? 0

Very satisfied

Satisfied

Neither satisfied nor dissatisfied

Dissatisfied

Very dissatisfied

(Please comment)

* 6. What were the three things most important to you about your baby’s

anesthetic? 0

Avoiding low blood pressure during the operation

Avoiding a breathing tube after surgery

Good pain relief after surgery

Avoiding low oxygen levels or breathing problems after surgery

Minimising impact on my baby's brain

Environmental impact of the anesthetic technique

Other, (please comment)

* 7. Thinking back, are there any questions you wish you had asked about your

baby’s anesthetist?

No

Yes (please comment)

* 8. We may do a further more in depth survey depending on the results of this

one. If you would be happy to be contacted please leave either a phone number

or email address.... 0

Name

Email Address

Phone Number

**Further information regarding the parent and carer survey.**

For parents and carers on infants who had their surgery at the Women’s and Children’s Hospital, Adelaide, Australia was emailed to parents following phone consent. If there was no answer after two phone attempts, no further attempts were made. Hospital records were cross-checked to ensure that parents and carers were not contacted if the child had been recorded as deceased. The Miracle Babies Foundation is an Australia-wide organisation that aims to support preterm and sick newborns, their families and the hospitals that care for them. Parents Who Have Been There is a registered not-for-profit charity consisting of Adelaide parents who had babies born preterm or unwell requiring Neonatal Care and offers support to parents and families throughout the neonatal journey, in hospital and beyond. By utilising these three methods of dissemination the aim was to obtain a larger number of responses, whilst still disseminating the survey only to the target population.
